# Assessment of renal hemodynamic toxicity of fluid challenge with 0.9% NaCl compared to balanced crystalloid (PlasmaLyte^®^) in a rat model with severe sepsis

**DOI:** 10.1186/s13613-017-0286-1

**Published:** 2017-06-14

**Authors:** Pierre-Yves Olivier, François Beloncle, Valérie Seegers, Maher Tabka, Mathilde Renou de La Bourdonnaye, Alain Mercat, Paul Cales, Daniel Henrion, Peter Radermacher, Lise Piquilloud, Nicolas Lerolle, Pierre Asfar

**Affiliations:** 1 CHU d’Angers, 4 rue Larrey, 49000 Angers, France; 2 Medical Intensive Care Department, University Hospital, Angers, France; 3 BNMI Laboratory, CNRS UMR 6214—INSERM U1083, Angers University, Angers, France; 4 Statistical Department, University Hospital, Angers, France; 5 Faculté de médecine d’Angers, 4 rue haute de reculée, 49000 Angers, France; 6 HIFIH, UPRE-EA 3859, SFR 4208, LUNAM University, Angers, France; 7 Universitätsklinikum, Ulm Helmholtzstr. 8/1, 89081 Ulm, Germany; 8 Institut für Anästhesiologische Pathophysiologie und Verfahrensentwicklung, Universitätsklinikum, Ulm, Germany

**Keywords:** Sepsis, Shock, Acute kidney injury, Hemodynamics, Microcirculation, Sodium chloride, PlasmaLyte, Crystalloid, Balanced crystalloid

## Abstract

**Background:**

According to international guidelines, volume expansion with crystalloids is the first-line treatment for hemodynamic management in patients with severe sepsis or septic shock. Compared to balanced crystalloids, 0.9% sodium chloride (0.9% NaCl) induces hyperchloremia and metabolic acidosis and may alter renal hemodynamics and function. We compared the effects of 0.9% NaCl to a less chloride-concentrated fluid, PlasmaLyte^®^ (PL) in targeted fluid resuscitation in a randomized, double-blind controlled study in an experimental model of severe sepsis in rats.

**Results:**

A sepsis with hypotension was induced by cecal ligature and puncture (CLP) in 40 male Wistar rats (20 for each crystalloid). Rats received fluid resuscitation over a period of 200 min for a targeted mean arterial pressure of 90 mm Hg. Animals received similar volumes of 0.9% NaCl or PL. Unlike PL-resuscitated rats, 0.9% NaCl-resuscitated rats experienced hyperchloremia and metabolic acidosis, whereas systemic hemodynamics, renal hemodynamics and renal function were not significantly different between both groups.

**Conclusion:**

In our model of rats with severe sepsis resuscitated with large amounts of crystalloids, 0.9% NaCl-induced hyperchloremic acidosis, but balanced crystalloid did not improve systemic and renal hemodynamics or renal function.

**Electronic supplementary material:**

The online version of this article (doi:10.1186/s13613-017-0286-1) contains supplementary material, which is available to authorized users.

## Background

Arterial hypotension in patients with septic shock and severe sepsis, related to hypovolemia and arterial vasodilation, leads to organ hypoperfusion and ultimately to organ failure. According to the most recent guidelines of the Surviving Sepsis Campaign (SSC), the first-line hemodynamic treatment is to restore blood volume by increasing cardiac preload with crystalloids [1]. The type of crystalloids is not specified, and especially if non-balanced (i.e., 0.9% sodium chloride) or balanced crystalloids should be preferentially used.

The sodium concentration in 0.9% sodium chloride (0.9% NaCl) is close to the sodium plasma concentration (154 vs 135–145 mmol/L, respectively), whereas compared to the chloride concentration in plasma, the 0.9% NaCl chloride concentration is higher (95–105 vs 154 mmol/L, respectively). Hence, when large amounts of 0.9% NaCl are infused in patients with septic shock, a high load of chloride is infused, and both hyperchloremia and subsequently metabolic acidosis may occur as reported in animal models, healthy volunteers and postoperative patients [2–4]. The mechanisms of hyperchloremic-induced acidosis combine the reduction of the anion gap [5] and the dilution of natural proton buffers (albumin and bicarbonates) [6]. Metabolic acidosis is not reported with so-called balanced crystalloid solutions, characterized by a chloride concentration closer to plasma chloride concentration, e.g., Ringer Lactate^®^ (110 mmol/L) or PlasmaLyte^®^ (PL) (98 mmol/L).

The clinical consequences of the metabolic perturbations related to 0.9% NaCl administration are unclear. Several animal studies suggest renal toxicity of hyperchloremic acidosis which leads to renal blood flow and renal filtration rate decrease [7–9], as well as inflammatory and hemodynamic effects [9, 10]. However, the clinical relevance of these models is debatable because of the non-physiological nature of the experimental studied systems (including isolated nephron or deinnervated kidney) and intervention (as infusion of very high concentration of chloride or hydrochloric acid).

A study in healthy volunteers also reported a decrease in renal blood flow after infusion of 0.9 NaCl compared to PL [11]. In patients, potential renal toxicity of hyperchloremic acidosis induced by 0.9% NaCl was reported in two cohort studies [15, 16], but was not confirmed by two small controlled studies [12, 13]. The most recent, large multi-center, controlled study in intensive care units, aimed at comparing 0.9% NaCl to PL used as fluid (SPLIT study [16]), did not report significantly different clinical outcomes between balanced and non-balanced crystalloids. However, the authors and editorialist [17] raised a major limitation of this study, inasmuch as SPLIT study only included a small proportion of patients who were at high risk of acute kidney injury or who required large volumes of intravenous fluids, such as patients with septic shock.

Conclusive data are hence lacking. Therefore, according to the SPLIT investigators’ recommendations, we aimed at comparing the renal effects of 0.9% NaCl versus balanced crystalloids in a clinically relevant model of fluid-resuscitated severe sepsis induced by cecal ligature and puncture in rats. In order to understand the putative toxic effect of chloride, we paid special attention to the relationship between systemic hemodynamics, renal blood flow and renal function.

## Methods

### Experimental model

This protocol received veterinary authority approval (Authorization notification No. 01008.02 from the French Ministry of Higher Education and Research).

We designed a randomized, blind, controlled, experimental prospective study using male Wistar rats (weight: 250–350 g) to compare 0.9% NaCl (Cl [154 mmol/L]) and PL (Cl [98 mmol/L]) used as resuscitation fluids in rats with severe sepsis induced by cecal ligature and puncture.

### Surgical preparation

Induction of anesthesia was performed with isoflurane (Piramal Healthcare Inc., Mumbai, India), followed by an intraperitoneal injection of thiopental (Rotex Medica Inc., Trittau, Germany) and ketamine (Renaudin Inc., Paris, France). After additional local anesthesia on the surgical sites (described below), rats were placed on a heating support. Then, they were tracheotomized, ventilated and perfused with a central vein catheter inserted via the left femoral vein. Mean arterial pressure was continuously monitored by a left femoral arterial catheter. The left renal and carotid arteries were isolated, and transit time ultrasound Doppler probes (Transonic System Inc., Ithaca, USA) were placed in contact with them. The bladder was cannulated. Finally, a cecal ligature and puncture (CLP) was performed to induce sepsis. After this surgical preparation was completed (T0, end of preparation), the rats were randomized to 0.9% NaCl or balanced crystalloid (PL) groups.

### Blinding and randomization methods

Before the start of the experiment, 0.9% NaCl and PL were sampled in 40 unmarked kits (20 of each fluid) containing 100 mL of 0.9% NaCl or PL and 10 mL of inulin diluted in 0.9% NaCl or PL (1 mg/mL). Kits were numbered randomly and supplied to the investigator who was blinded for the group assignment.

### Sepsis induction and monitoring

**Fig. 1:**
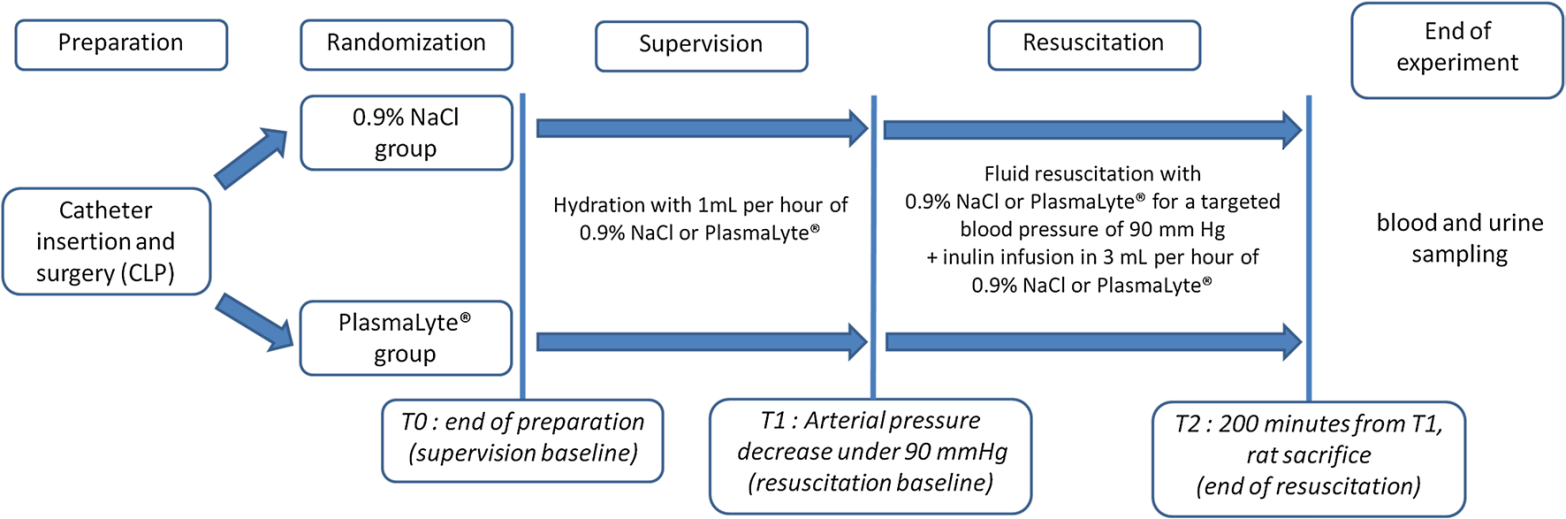
Experimental design. After catheter insertion and surgical CLP, rats were randomized into the 0.9% NaCl or PlasmaLyte^®^ groups. After randomization (T0, supervision baseline), rats were hydrated with the corresponding crystalloid and were monitored until arterial pressure dropped below 90 mm Hg (T1, end of supervision, resuscitation* baseline*). At that time, they received the allocated crystalloid for 200 min for a targeted blood pressure of 90 mmHg. After 200 min, the animals were killed and their urine and plasma were sampled (T2, end of resuscitation)

After sepsis induction by CLP, depending on the randomization arm, the rats were hydrated with 1 mL/H of 0.9% NaCl or PL. Mean arterial pressure was monitored every 10 min until arterial pressure dropped below 90 mm Hg (T1, resuscitation baseline, T0 to T1 = supervision). The rats were then considered hypotensive, and depending on their randomization arm, continuous infusion was increased to 3 mL/H (base infusion). In addition, animals received additional fluid challenges targeted to maintain a mean arterial pressure at 90 mm Hg. The rats were monitored for 200 min from the T1 (T2, end of resuscitation, T1 to T2 = resuscitation) (Fig. 1).

### Data collection

Heart rate, mean arterial blood pressure, pressure in the inferior vena cava considered as a surrogate of central venous pressure, left carotid and left renal arteries blood flow, core temperature and urine output were recorded every 10 min.

Doppler probes on left renal and left carotid arteries allowed a continuous monitoring of blood flows (displayed as a numeric mean value in milliliter per minute). The quality of signal was assessed by the adequate pulsatility and the absence of short-term lability (the lack of these criteria led to probe adjustments). The absence of contralateral stenosis, dilatation or renal malformation was controlled after animal killing.

Renal microcirculation was evaluated by measuring velocity in cortical capillaries with Side Dark Field Camera movies (Micro Vision Medical Inc., Amsterdam, the Netherlands). Videos were sampled at T1, T1 + 90 min and T1 + 180 min. Analyses were performed off-line with AVA software (Micro Vision Medical Inc., Amsterdam, the Netherlands). Twenty capillaries (5 capillaries per quadrant) per video were analyzed, and velocities were determined on, at least, three blood cells, as previously described [18].

Renal function was assessed by measuring inulin and creatinine clearance. We used FTIC-labeled inulin (Sigma Inc., Paris, France) diluted in the 3-mL/H base infusion of 0.9% NaCl or PL (depending on the randomization arm), with a 1-mg/mL concentration. Clearance was calculated at the end of the experiment according to the formulae: *U* × *V*/*P* (*U* for urine concentration, *V* for urine volume/time and *P* for plasma concentration). We used for inulin blood concentration measurement blood sampled at the end of resuscitation and for inulin urine concentration measurement urine sampled from the last hour of resuscitation. The urine volume corresponded to the volume sampled during the last hour of resuscitation. Rats of similar age and weight were supposed to have similar and normal baseline renal function. To reduce blood sampling volume before a severe surgical stress and to assess RIFLE score, we measured baseline blood creatinine in ten training test rats and considered the mean creatinine value for the RIFLE calculation.

Urine was collected at T0 and T2 to measure the concentration of chloride, sodium, potassium, blood urea nitrogen (BUN) and creatinine. Arterial blood was sampled at T2 to measure chloride, sodium, potassium, BUN, creatinine, hematocrit, proteins, pH, phosphate, calcium, bicarbonates, PaO_2_ and PaCO_2_.

### Data analysis

Quantitative data are expressed as the mean and standard deviation. A nonparametric Mann–Whitney test was used for intergroup comparisons. For repeated measurements, we used a mixed linear regression model [19]. All statistics were calculated with R software 3.2.2 (free software developed by R Core Team (2008) for the R Foundation for Statistical Computing, Vienna, Austria) and Nlme R package version 3.1-122 (free software developed by Pinheiro J, Bates D, DebRoy S, Sarkar D and R Core Team (2015) for the R Foundation for Statistical Computing, Vienna, Austria). All tests were bilateral, and a *p* value of <0.05 was considered statistically significant.

## Results

### Baseline characteristics

Baseline characteristics, at T0 (weight, mean arterial pressure, carotid and renal blood flows, central venous pressure and initial urines analysis), were similar in the groups (Table 1).

**Table 1:** Baseline characteristics

	0.9% NaCl *n* = 20	PlasmaLyte^®^ *n* = 20	*p*
Weight (g)	291 ± 24	293 ± 25	0.80
Mean arterial pressure (mm Hg)			
T0	129 ± 9	126 ± 9	0.30
T1	88 ± 2	86 ± 3	0.17
Mean carotid arterial blood flow (mL/min)			
T0	2.9 ± 0.9	3.0 ± 1.0	0.61
T1	1.8 ± 0.8	1.6 ± 0.5	0.48
Mean renal arterial blood flow (mL/min)			
T0	2.6 ± 1.3	2.1 ± 1.0	0.27
T1	3.0 ± 1.1	2.4 ± 1.2	0.19
Hypotension occurrence delays (min)	176 ± 91	177 ± 74	0.96
T0 urine concentrations (mmol/L)			
Sodium	93 ± 29	88 ± 37	0.52
Chloride	162 ± 56	148 ± 70	0.5
Potassium	172 ± 68	164 ± 66	0.77
BUN	516 ± 144	510 ± 191	0.82
Creatinine	6.0 ± 1.9	6.0 ± 2.9	0.63

### Sepsis induction

After cecal ligation and puncture, hypotension occurred within similar time frames in the two groups (205 ± 40 min in the 0.9% NaCl group vs 190 ± 32 min in the PL group, *p* = 0.85). Hemodynamics at hypotension time were similar in both groups (Table 1).

Data are presented as mean and standard deviation. There was no significant difference of baseline characteristics between 0.9% NaCl and PlasmaLyte^®^ at the end of preparation or at the time of hypotension occurrence. BUN = blood urea nitrogen. T0: end of preparation and supervision baseline. T1: occurrence of hypotension and resuscitation baseline.

### Resuscitation and systemic hemodynamics

**Fig. 2:**
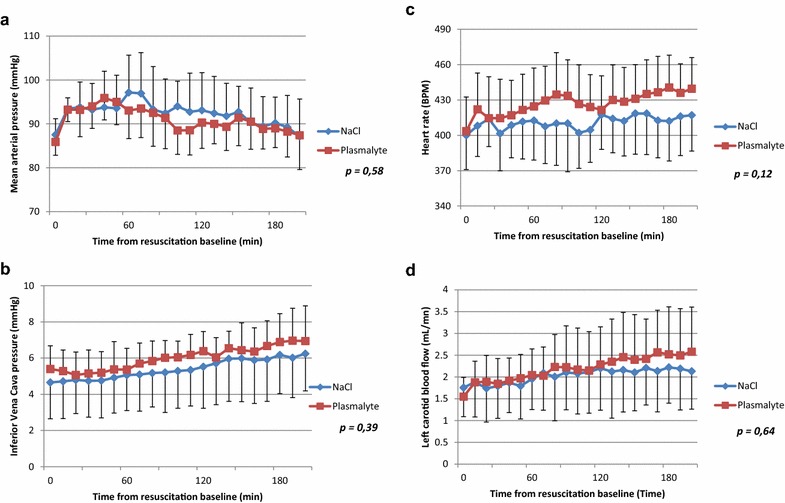
Systemic hemodynamics during resuscitation. All data are presented as mean and standard deviation. Statistics were performed with a linear regression model. Mean arterial pressure is reported (**a**) and was maintained at approximately 90 mm Hg in both groups. Inferior vena cava (**b**), heart rate (**c**), left carotid blood flow (**d**) were no significantly different between the two groups

During the 200-min resuscitation period, mean arterial blood pressure was successfully maintained above 90 mm Hg during at least 90% of resuscitation time in both groups (Fig. 2). Heart rate, central venous pressure and carotid blood flow increased significantly during the resuscitation period without intergroup differences.

For a similar effect on systemic hemodynamic parameters, both groups required the same fluid volume (20 mL average in addition to 3 mL/H base infusion) to target a mean arterial pressure of 90 mm Hg. The indirect markers of volume expansion such as hematocrit and plasma protein concentration were not significantly different (Table 2).

**Table 2:** Plasma, arterial blood gas and urine data at 200 min postresuscitation

	0.9% NaCl *n* = 20	PlasmaLyte^®^ *n* = 20	*p*
Total fluid requirements (mL) during resuscitation	23 ± 11	23 ± 10	0.99
Plasma at T2			
Sodium (mmol/L)	141 ± 2	138 ± 3	0.017
Chloride (mmol/L)	117 ± 3	104 ± 3	<0.001
Potassium (mmol/L)	6.0 ± 0.6	5.3 ± 0.6	0.004
BUN (mmol/L)	12 ± 3	12 ± 4	0.77
Creatinine (µmol/L)	34 ± 7	36 ± 6	0.27
Phosphate (mmol/L)	3.5 ± 0.5	3.6 ± 0.5	0.36
Calcium (mmol/L)	1.9 ± 0.1	1.9 ± 0.2	0.64
Osmolarity (mmol/L)	306 ± 8	299 ± 8	0.027
Hematocrit (%)	44 ± 6	48 ± 4	0.16
Plasma protein (g/L)	27 ± 3	29 ± 3	0.33
Arterial blood gas			
pH	7.34 ± 0.05	7.44 ± 0.04	<0.001
Bicarbonate (mmol/L)	20 ± 2	26 ± 2	<0.001
PaCO_2_ (mm Hg)	38 ± 5	39 ± 5	0.62
Urine at T2			
Urine output during resuscitation (mL)	1.7 ± 0.4	2.5 ± 0.6	0.002
Sodium (mmol/L)	52 ± 28	41 ± 15	0.4
Chloride (mmol/L)	182 ± 56	26 ± 6	<0.001
Potassium (mmol/L)	168 ± 48	175 ± 54	0.75
BUN (mmol/L)	533 ± 196	383 ± 161	0.066
Creatinine (mmol/L)	6.0 ± 2.0	4.2 ± 1.3	0.019
Sodium/potassium ratio	0.38 ± 0.24	0.29 ± 0.17	0.44
Measured clearance at T2			
Inulin (mL/min)	5.2 ± 2.9	4.4 ± 1.9	0.49
Creatinine (mL/min)	2.3 ± 1.1	2.3 ± 0.8	0.95

Data are presented as mean and standard deviation. Rats in the 0.9% NaCl group had a significant lower arterial pH and HCO^3−^ concentration, higher plasma Na^+^, Cl^−^ and K^+^ concentrations and higher plasma osmolarity. There was no significant difference in renal inulin and creatinine clearance between the two groups. BUN = blood urea nitrogen. T2: end of resuscitation. Osmolarity was calculated as follows:

$$\left( {{\text{sodium}} + {\text{potassium}}} \right) \times 2 + {\text{BUN}}.$$

### Metabolic data

At the end of the experiment, arterial pH and bicarbonates were significantly lower in the 0.9% NaCl group than in PL-treated rats, whereas PaCO_2_ was similar in both groups. Sodium, chloride and potassium plasma concentrations were significantly higher in the 0.9% NaCl group compared to the PL group. The other assayed ions did not differ between the groups (Table 2).

At the end of the experiment, ion urine concentrations were similar in both groups, except for chloride, which was significantly higher in the 0.9% NaCl-treated rats (Table 2).

### Renal hemodynamics

**Fig. 3:**
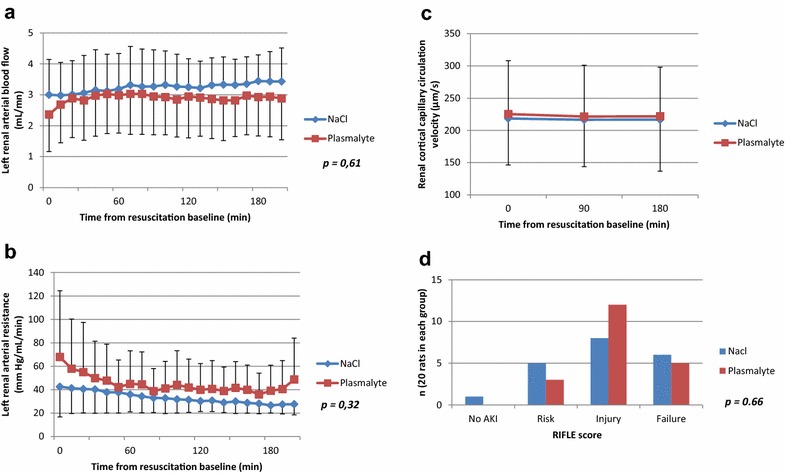
Renal hemodynamic and renal function during resuscitation. All data are presented as mean and standard deviation; *p* values were obtained using a mixed linear regression model for **a** and **b** and using the Mann–Whitney test for **c** and **d**. There was no significant difference between the two groups in the left arterial renal rate measured by TTU probes (**a**),* left* renal arterial resistance (**b**) and renal cortical capillary velocity (**c**) measured by Side Dark Field Camera (*p* = 0.41 at resuscitation* baseline* time, *p* = 0.54 at 90 min after resuscitation baseline and *p* = 0.51 at 180 min after resuscitation* baseline*). Distribution in the RIFLE classification (**d**) (based on creatinine plasma concentration, inulin clearance and creatinine clearance) was similar in both groups as shown in **a** (*p* = 0.66 for all classes, *p* = 0.34 for RIFLE score 0, *p* = 0.44 for RIFLE score 1, *p* = 0.22 for RIFLE score 2 and *p* = 0.74 for RIFLE score 3)

Baseline renal arterial blood flow and renal arterial resistances were similar in both groups and remained similar at the end of the experiment. Cortical renal velocities remained stable and similar in the both groups (Fig. 3).

### Renal function data

Urine output was significantly higher in the PL group compared to the 0.9% NaCl group (1.65 ± 0.17 vs 2.4 ± 0.28 mL, respectively, *p* = 0.002), but urine was more concentrated in the 0.9% NaCl group (creatinine concentration: 6.2 ± 0.88 vs 4.45 ± 0.57 mmol/L, respectively, *p* = 0.019). Renal function was similarly impaired in both groups, with no significant difference in creatinine and BUN plasma concentrations, inulin and creatinine clearance (Table 2), as well as RIFLE score distribution (Fig. 3).

## Discussion

In this experimental study, we aimed at comparing the short-term effects of 0.9% NaCl and PL, a balanced crystalloid, used for fluid resuscitation in hypotensive rats with sepsis, on systemic and renal hemodynamics. To summarize, we found that both crystalloids had similar effects on systemic and renal hemodynamics and on renal function despite the occurrence of hyperchloremic acidosis in 0.9% NaCl group.

As expected, the NaCl 0.9% group showed hyperchloremic acidosis. The occurrence of hyperkalemia, despite the absence of potassium load in 0.9% NaCl group versus a 5-mmol/L load in PL group, was most likely related to acidosis. O’Malley et al. reported a similar effect, in patients with renal graft [13], and hyperkalemia led to premature stop of the trial. Hypernatremia and hyperosmolarity could be explained by the massive load of sodium-rich fluid in 0.9% NaCl.

Conversely, lower urine output in 0.9% NaCl group was not expected. Retrospectively, we hypothesized that the lower osmotic load induced by PL infusion may have favored a lower secretion of antidiuretic hormone, as suggested by Williams et al. [11] and Reid et al. [20], that reported similar higher urine output in healthy volunteers (of note, in these studies, osmotic gap between used balanced solution, Hartmann’s solution and 0.9% NaCl was higher than between PL and 0.9% NaCl). The second hypothesis involves a specific action of chloride, leading to a decrease in glomerular filtration rate, as suggested by Wilcox [7] or Ren et al. [8]. However, our hemodynamic and functional renal data did not provide evidence for this mechanism. Finally, due to the short-term design of our study, we cannot exclude that a similar urine output would have been reported in both groups with a longer follow-up.

Tubuloglomerular feedback is thought to play a central role in renal chloride toxicity. In isolated kidneys or nephrons, hyperchloremia and reabsorption of large amounts of chloride by the *macula densa* activate tubular–glomerular feedback as well as the renin angiotensin system, inducing a vasoconstriction of both afferent and efferent glomerular arteries, which, in turn, decreases both renal blood flow and glomerular filtration rate [7–9]. The decrease in renal blood flow consecutive to 0.9% NaCl-induced hyperchloremic acidosis was also reported in healthy volunteers [11].

All these animal studies reported an early renal vasoconstrictor effect of chloride but have used questionable models (isolated nephron, deinnervated kidney, etc.). In order to explore this specific issue of renal hemodynamic effect of chloride, in a more clinically relevant model, we have designed a severe septic fluid-resuscitated model at high risk of renal injury and requiring large crystalloid amounts. By design, due to the severity of the model, it was not possible to assess the long-term effect of chloride renal toxicity. Our study adds new information combining entire animal, large sample for an animal study, large amounts of chloride, severe and pathophysiologically relevant model of sepsis and renal hemodynamics. Our study does not provide evidence for short-term renal vasoconstriction related to high chloride load both at macrocirculatory and at microcirculatory levels.

The study of delayed renal toxicity of chloride related to inflammatory mechanisms was not our purpose, and we do not deny that these mechanisms are involved in renal chloride toxicity. On this topic, Zhou et al. reported a beneficial effect of PL in a less severe model of sepsis in awake rats. In this study, rats were infused with smaller amounts of crystalloids for a longer time [21]. PlasmaLyte^®^ infusion was associated with a better renal function and an attenuated inflammatory response. Our results contrast with Zhou et al. on renal function alteration in the PL group, although our rats seem to present similar metabolic disturbances in the 0.9% NaCl group. In our experimental study, we infused the fourfold amount of crystalloids within a shorter time in anesthetized and ventilated rats. In addition, contrary to Zhou et al., we targeted fluid resuscitation according to mean arterial pressure. The impact of these different experimental designs and severity of hypotension remains speculative. Interestingly, in a more severe hypotensive sepsis model induced by endotoxin injection, Ergin et al. [22] reported no difference either between a balanced experimental crystalloid (Cl^−^[119 mmol/L]) and 0.9% NaCl in renal function and renal blood flow.

The clinical relevance of high chloride load and hyperchloremic acidosis remains, respectively, unclear. In surgical patients, the use of 0.9% NaCl, but not balanced crystalloid solutions, was associated with hyperchloremia and metabolic acidosis, however, without significant effects on morbidity, mortality and renal function [14, 15]. These results have led some authors to question the clinical consequences of hyperchloremic acidosis and to challenge the use of balanced crystalloids [23]. Two cohort studies, one retrospective [12] and the other with a before-and-after two-period design [13], fueled the debate in 2012 as they have reported a significant reduction in acute renal failure in postoperative and intensive care patients treated with balanced solutions. These findings were not confirmed by the randomized SPLIT trial [16]. It was argued in the SPLIT trial that the severity of patients mirrored by the mortality rate (7.6 and 8.6% in the PlasmaLyte^®^ and saline groups, respectively) and low fluid requirements was low (2.7 vs 2.6 l, respectively) and may have explained the absence of beneficial effects of PlasmaLyte^®^. However, two other recent large controlled randomized studies, with higher chloride amounts, in patients presenting high risk of renal acute injury, comparing 3% NaCl to 0.9% NaCl in patients in septic shock for the first one and comparing 0.9% NaCl to PL in patients after cardiac surgery for the second one, reported both hyperchloremic acidosis in patients receiving higher load of chloride but did not reported any detrimental renal effect for these patients [24, 25].

Our study has several limitations. The short duration of our follow-up does not allow us to rule out a delayed detrimental effect. When we designed this study, the available animal data suggested a very early detrimental effect of chloride, supposed to be mediated by short-term mechanisms such as tubuloglomerular feedback and/or renin angiotensin system. Therefore, we designed our study to investigate the early hemodynamic effects and the potential renal functional consequences. We cannot exclude a delayed effect of chloride, but, to the best of our knowledge, there is no clear argument suggesting that this potential delayed effect would not have been preceded by a hemodynamic effect. Zhou et al. investigated the delayed effects of chloride and suggested a delayed toxicity of chloride acidosis, but did not provide data on renal hemodynamic during early phase of resuscitation. However, our study allows the pragmatic conclusion that putative detrimental effect of 0.9% NaCl is neither induced by renal macrocirculatory nor by microcirculatory disturbances. In addition, our study is limited by the absence of cardiac output monitoring, even though this is a major determinant of renal blood flow [26]. As it is a highly invasive surgical procedure in rats, we therefore used carotid blood flow as a surrogate for cardiac output [27]. The risk of bias is also limited as we directly measured renal blood flow in order to check an unexpected alteration in renal blood flow between the two crystalloids. Last limitation is the lack of in vivo assessment of glomerular arteries due to technical reasons, but we measured the cortical microcirculatory velocities which mirror postglomerular microcirculatory blood flow. Therefore, a change in upstream blood flow (i.e., afferent and/or efferent glomerular arteries) should have been detected.

## Conclusion

In our model of rats with severe sepsis resuscitated with large amounts of crystalloids, 0.9% NaCl-induced hyperchloremic acidosis, but balanced crystalloid did not improve systemic and renal hemodynamics or renal function.

## Additional file

**Additional file 1**. **Study Database.** Each column corresponds to one animal. Time is indicated in minutes, other units are mentioned in first, second or third column. Empty box corresponds to lacking data for technical reason (unworkable biologic sampling or video).

## Abbreviations

BUN: blood urea nitrogen
CLP: cecal ligature and puncture
PL: PlasmaLyte^®^
